# Supplementary material for: Antibody responses to SARS-CoV-2 in patients with differing severities of coronavirus disease 2019
Source: PLoS One. 2020 Oct 9;15(10):e0240502. doi: 10.1371/journal.pone.0240502 (PMC7546485; doi:10.1371/journal.pone.0240502)
Supplement: S1 Table — (DOCX) [file pone.0240502.s001.docx]

**Antibody Responses to SARS-CoV-2 in Coronavirus Diseases 2019 Patients with Different Severity**

**S1 Table: Raw data of control group**

| **No** | **Details** | **ELISA_IgA (OD Ratio)** | **Interpretation (0=neg, 1=pos)** | **ELISA_IgG (OD Ratio)** | **Interpretation (0=neg, 1=pos)** |  | **ELISA_IgA (OD Ratio)** | **ELISA_IgG (OD Ratio)** |
| --- | --- | --- | --- | --- | --- | --- | --- | --- |
| 1 | Healthy donor | 0.57 | 0 | 0.13 | 0 |  |  |  |
| 2 | Healthy donor | 0.47 | 0 | 0.18 | 0 |  |  |  |
| 3 | Healthy donor | 0.39 | 0 | 0.19 | 0 |  |  |  |
| 4 | Healthy donor | 0.69 | 0 | 0.19 | 0 |  |  |  |
| 5 | Healthy donor | 0.12 | 0 | 0.21 | 0 |  |  |  |
| 6 | Healthy donor | 1.32 | 1 | 0.18 | 0 | Repeat after 2 months | 1.26 | 0.19 |
| 7 | Healthy donor | 0.44 | 0 | 0.52 | 0 |  |  |  |
| 8 | Healthy donor | 0.26 | 0 | 0.17 | 0 |  |  |  |
| 9 | Healthy donor | 0.88 | 0 | 0.35 | 0 | Repeat after 2 months | 0.97 | 0.37 |
| 10 | Healthy donor | 0.29 | 0 | 0.17 | 0 |  |  |  |
| 11 | Healthy donor | 0.39 | 0 | 0.17 | 0 |  |  |  |
| 12 | Healthy donor | 0.40 | 0 | 0.17 | 0 |  |  |  |
| 13 | Healthy donor | 0.33 | 0 | 0.14 | 0 |  |  |  |
| 14 | Healthy donor | 0.80 | 0 | 0.23 | 0 | Repeat after 2 months | 0.75 | 0.22 |
| 15 | Healthy donor | 0.95 | 0 | 0.16 | 0 | Repeat after 2 months | 0.97 | 0.17 |
| 16 | Healthy donor | 0.80 | 0 | 0.13 | 0 | Repeat after 2 months | 0.84 | 0.19 |
| 17 | Healthy donor | 0.22 | 0 | 0.12 | 0 |  |  |  |
| 18 | Healthy donor | 0.23 | 0 | 0.22 | 0 |  |  |  |
| 19 | Healthy donor | 0.83 | 0 | 0.22 | 0 | Repeat after 2 months | 0.92 | 0.31 |
| 20 | Healthy donor | 0.66 | 0 | 0.23 | 0 |  |  |  |
| 21 | Healthy donor | 0.58 | 0 | 0.19 | 0 |  |  |  |
| 22 | Healthy donor | 0.45 | 0 | 0.21 | 0 |  |  |  |
| 23 | Healthy donor | 0.69 | 0 | 0.21 | 0 |  |  |  |
| 24 | Healthy donor | 0.45 | 0 | 0.15 | 0 |  |  |  |
| 25 | Healthy donor | 0.20 | 0 | 0.21 | 0 |  |  |  |
| 26 | Healthy donor | 0.28 | 0 | 0.15 | 0 |  |  |  |
| 27 | Healthy donor | 0.64 | 0 | 0.23 | 0 |  |  |  |
| 28 | Healthy donor | 0.34 | 0 | 0.14 | 0 |  |  |  |
| 29 | Healthy donor | 0.35 | 0 | 0.14 | 0 |  |  |  |
| 30 | Healthy donor | 0.53 | 0 | 0.52 | 0 |  |  |  |
| 31 | Healthy donor | 0.40 | 0 | 0.17 | 0 |  |  |  |
| 32 | Healthy donor | 0.92 | 0 | 0.36 | 0 | Repeat after 2 months | 1.11 | 0.44 |
| 33 | Healthy donor | 0.47 | 0 | 0.15 | 0 |  |  |  |
| 34 | Healthy donor | 0.28 | 0 | 0.21 | 0 |  |  |  |
| 35 | Healthy donor | 0.28 | 0 | 0.15 | 0 |  |  |  |
| 36 | Healthy donor | 0.27 | 0 | 0.17 | 0 |  |  |  |
| 37 | Healthy donor | 0.36 | 0 | 0.15 | 0 |  |  |  |
| 38 | Healthy donor | 0.78 | 0 | 0.15 | 0 |  |  |  |
| 39 | Healthy donor | 0.37 | 0 | 0.15 | 0 |  |  |  |
| 40 | Healthy donor | 0.18 | 0 | 0.21 | 0 |  |  |  |
| 41 | Healthy donor | 0.24 | 0 | 0.15 | 0 |  |  |  |
| 42 | Healthy donor | 0.47 | 0 | 0.31 | 0 |  |  |  |
| 43 | Healthy donor | 0.46 | 0 | 0.16 | 0 |  |  |  |
| 44 | Healthy donor | 0.32 | 0 | 0.18 | 0 |  |  |  |
| 45 | Healthy donor | 0.27 | 0 | 0.16 | 0 |  |  |  |
| 46 | Healthy donor | 0.25 | 0 | 0.14 | 0 |  |  |  |
| 47 | Healthy donor | 0.37 | 0 | 0.24 | 0 |  |  |  |
| 48 | Healthy donor | 0.87 | 0 | 0.26 | 0 |  |  |  |
| 49 | Healthy donor | 0.42 | 0 | 0.19 | 0 |  |  |  |
| 50 | Healthy donor | 0.06 | 0 | 0.24 | 0 |  |  |  |
| 51 | Healthy donor | 0.25 | 0 | 0.22 | 0 |  |  |  |
| 52 | Healthy donor | 0.78 | 0 | 0.42 | 0 |  |  |  |
| 53 | Healthy donor | 0.26 | 0 | 0.21 | 0 |  |  |  |
| 54 | Healthy donor | 0.30 | 0 | 0.14 | 0 |  |  |  |
| 55 | Healthy donor | 0.42 | 0 | 0.18 | 0 |  |  |  |
| 56 | Healthy donor | 0.25 | 0 | 0.18 | 0 |  |  |  |
| 57 | Healthy donor | 0.37 | 0 | 0.17 | 0 |  |  |  |
| 58 | Healthy donor | 0.42 | 0 | 0.18 | 0 |  |  |  |
| 59 | Healthy donor | 0.61 | 0 | 0.44 | 0 |  |  |  |
| 60 | Healthy donor | 0.46 | 0 | 0.17 | 0 |  |  |  |
| 61 | Healthy donor | 0.51 | 0 | 0.20 | 0 |  |  |  |
| 62 | Healthy donor | 0.45 | 0 | 0.13 | 0 |  |  |  |
| 63 | Healthy donor | 0.34 | 0 | 0.14 | 0 |  |  |  |
| 64 | Healthy donor | 0.20 | 0 | 0.18 | 0 |  |  |  |
| 65 | Healthy donor | 0.78 | 0 | 0.17 | 0 |  |  |  |
| 66 | Healthy donor | 0.42 | 0 | 0.24 | 0 |  |  |  |
| 67 | Healthy donor | 0.37 | 0 | 0.16 | 0 |  |  |  |
| 68 | Healthy donor | 0.28 | 0 | 0.13 | 0 |  |  |  |
| 69 | Healthy donor | 2.83 | 1 | 0.50 | 0 |  |  |  |
| 70 | Healthy donor | 0.24 | 0 | 0.35 | 0 |  |  |  |
| 71 | Healthy donor | 0.49 | 0 | 0.17 | 0 |  |  |  |
| 72 | Healthy donor | 0.22 | 0 | 0.17 | 0 |  |  |  |
| 73 | Healthy donor | 0.44 | 0 | 0.47 | 0 |  |  |  |
| 74 | Healthy donor | 0.29 | 0 | 0.27 | 0 |  |  |  |
| 75 | Healthy donor | 0.28 | 0 | 0.16 | 0 |  |  |  |
| 76 | Healthy donor | 0.56 | 0 | 0.20 | 0 |  |  |  |
| 77 | Healthy donor | 0.22 | 0 | 0.30 | 0 |  |  |  |
| 78 | Healthy donor | 0.12 | 0 | 0.12 | 0 |  |  |  |
| 79 | Healthy donor | 0.37 | 0 | 0.26 | 0 |  |  |  |
| 80 | Healthy donor | 0.68 | 0 | 0.13 | 0 |  |  |  |
| 81 | Healthy donor | 0.19 | 0 | 0.14 | 0 |  |  |  |
| 82 | Healthy donor | 0.51 | 0 | 0.20 | 0 |  |  |  |
| 83 | Healthy donor | 0.19 | 0 | 0.22 | 0 |  |  |  |
| 84 | Healthy donor | 0.26 | 0 | 0.17 | 0 |  |  |  |
| 85 | Healthy donor | 0.13 | 0 | 0.21 | 0 |  |  |  |
| 86 | Healthy donor | 0.25 | 0 | 0.16 | 0 |  |  |  |
| 87 | Healthy donor | 0.12 | 0 | 0.13 | 0 |  |  |  |
| 88 | Healthy donor | 0.20 | 0 | 0.16 | 0 |  |  |  |
| 89 | Healthy donor | 0.51 | 0 | 0.20 | 0 |  |  |  |
| 90 | Healthy donor | 0.30 | 0 | 0.32 | 0 |  |  |  |
| 91 | Healthy donor | 0.41 | 0 | 0.16 | 0 |  |  |  |
| 92 | Healthy donor | 0.26 | 0 | 0.28 | 0 |  |  |  |
| 93 | Healthy donor | 0.48 | 0 | 1.29 | 1 |  |  |  |
| 94 | Healthy donor | 0.44 | 0 | 0.16 | 0 |  |  |  |
| 95 | Healthy donor | 0.14 | 0 | 0.19 | 0 |  |  |  |
| 96 | Healthy donor | 0.33 | 0 | 0.37 | 0 |  |  |  |
| 97 | Healthy donor | 0.24 | 0 | 0.18 | 0 |  |  |  |
| 98 | Healthy donor | 0.56 | 0 | 0.23 | 0 |  |  |  |
| 99 | Healthy donor | 0.70 | 0 | 0.27 | 0 |  |  |  |
| 100 | Healthy donor | 0.62 | 0 | 0.29 | 0 |  |  |  |
| 101 | Healthy donor | 0.23 | 0 | 0.33 | 0 |  |  |  |
| 102 | Healthy donor | 0.18 | 0 | 0.17 | 0 |  |  |  |
| 103 | Patient under investigation SARS-CoV-2 PCR negative | 0.71 | 0 | 0.40 | 0 |  |  |  |
| 104 | Patient under investigation SARS-CoV-2 PCR negative | 0.56 | 0 | 0.13 | 0 |  |  |  |
| 105 | Patient under investigation SARS-CoV-2 PCR negative | 0.48 | 0 | 0.32 | 0 |  |  |  |
| 106 | Patient under investigation SARS-CoV-2 PCR negative | 0.38 | 0 | 0.47 | 0 |  |  |  |
| 107 | Patient under investigation SARS-CoV-2 PCR negative | 0.35 | 0 | 0.28 | 0 |  |  |  |
| 108 | Patient under investigation SARS-CoV-2 PCR negative | 0.22 | 0 | 0.25 | 0 |  |  |  |
| 109 | Patient under investigation SARS-CoV-2 PCR negative | 0.21 | 0 | 0.27 | 0 |  |  |  |
| 110 | Patient under investigation SARS-CoV-2 PCR negative | 0.37 | 0 | 0.27 | 0 |  |  |  |
| 111 | Patient under investigation SARS-CoV-2 PCR negative | 0.7 | 0 | 0.25 | 0 |  |  |  |
| 112 | Patient under investigation SARS-CoV-2 PCR negative | 0.61 | 0 | 0.28 | 0 |  |  |  |
| 113 | Patient under investigation SARS-CoV-2 PCR negative | 0.37 | 0 | 0.37 | 0 |  |  |  |
| 114 | Patient under investigation SARS-CoV-2 PCR negative | 0.29 | 0 | 0.23 | 0 |  |  |  |
| 115 | Patient under investigation SARS-CoV-2 PCR negative | 0.47 | 0 | 0.31 | 0 |  |  |  |
| 116 | Patient under investigation SARS-CoV-2 PCR negative | 1.88 | 1 | 0.56 | 0 |  |  |  |
| 117 | Patient under investigation SARS-CoV-2 PCR negative | 0.25 | 0 | 0.27 | 0 |  |  |  |
| 118 | Patient under investigation SARS-CoV-2 PCR negative | 0.23 | 0 | 0.39 | 0 |  |  |  |
| 119 | Patient under investigation SARS-CoV-2 PCR negative | 0.58 | 0 | 0.32 | 0 |  |  |  |
| 120 | Patient under investigation SARS-CoV-2 PCR negative | 0.35 | 0 | 0.32 | 0 |  |  |  |
| 121 | Patient under investigation SARS-CoV-2 PCR negative | 9.49 | 1 | 11.22 | 1 |  |  |  |
| 122 | Patient under investigation SARS-CoV-2 PCR negative | 6.11 | 1 | 1.37 | 1 | Repeat after 2 weeks | 0.45 | 0.17 |
| 123 | Patient under investigation SARS-CoV-2 PCR negative | 5.46 | 1 | 0.78 | 0 |  |  |  |
| 124 | Patient under investigation SARS-CoV-2 PCR negative | 1.31 | 1 | 0.48 | 0 | Repeat after 1 month | 0.25 | 0.18 |
| 125 | Patient under investigation SARS-CoV-2 PCR negative | 0.91 | 0 | 0.26 | 0 |  |  |  |
| 126 | Patient under investigation SARS-CoV-2 PCR negative | 0.90 | 0 | 0.41 | 0 |  |  |  |
| 127 | Patient under investigation SARS-CoV-2 PCR negative | 0.72 | 0 | 0.23 | 0 |  |  |  |
| 128 | Patient under investigation SARS-CoV-2 PCR negative | 0.62 | 0 | 0.22 | 0 |  |  |  |
| 129 | Patient under investigation SARS-CoV-2 PCR negative | 0.54 | 0 | 0.21 | 0 |  |  |  |
| 130 | Patient under investigation SARS-CoV-2 PCR negative | 0.52 | 0 | 0.18 | 0 |  |  |  |
| 131 | Patient under investigation SARS-CoV-2 PCR negative | 0.50 | 0 | 0.22 | 0 |  |  |  |
| 132 | Patient under investigation SARS-CoV-2 PCR negative | 0.50 | 0 | 0.20 | 0 |  |  |  |
| 133 | Patient under investigation SARS-CoV-2 PCR negative | 0.49 | 0 | 0.17 | 0 |  |  |  |
| 134 | Patient under investigation SARS-CoV-2 PCR negative | 0.47 | 0 | 0.21 | 0 |  |  |  |
| 135 | Patient under investigation SARS-CoV-2 PCR negative | 0.46 | 0 | 0.41 | 0 |  |  |  |
| 136 | Patient under investigation SARS-CoV-2 PCR negative | 0.44 | 0 | 0.37 | 0 |  |  |  |
| 137 | Patient under investigation SARS-CoV-2 PCR negative | 0.44 | 0 | 0.16 | 0 |  |  |  |
| 138 | Patient under investigation SARS-CoV-2 PCR negative | 0.40 | 0 | 0.26 | 0 |  |  |  |
| 139 | Patient under investigation SARS-CoV-2 PCR negative | 0.35 | 0 | 0.20 | 0 |  |  |  |
| 140 | Patient under investigation SARS-CoV-2 PCR negative | 0.33 | 0 | 0.23 | 0 |  |  |  |
| 141 | Patient under investigation SARS-CoV-2 PCR negative | 0.33 | 0 | 0.20 | 0 |  |  |  |
| 142 | Patient under investigation SARS-CoV-2 PCR negative | 0.33 | 0 | 0.17 | 0 |  |  |  |
| 143 | Patient under investigation SARS-CoV-2 PCR negative | 0.30 | 0 | 0.24 | 0 |  |  |  |
| 144 | Patient under investigation SARS-CoV-2 PCR negative | 0.30 | 0 | 0.28 | 0 |  |  |  |
| 145 | Patient under investigation SARS-CoV-2 PCR negative | 0.29 | 0 | 0.22 | 0 |  |  |  |
| 146 | Patient under investigation SARS-CoV-2 PCR negative | 0.28 | 0 | 0.21 | 0 |  |  |  |
| 147 | Patient under investigation SARS-CoV-2 PCR negative | 0.22 | 0 | 0.18 | 0 |  |  |  |
| 148 | Patient under investigation SARS-CoV-2 PCR negative | 0.19 | 0 | 0.22 | 0 |  |  |  |
| 149 | Patient under investigation SARS-CoV-2 PCR negative | 0.19 | 0 | 0.18 | 0 |  |  |  |
| 150 | Patient under investigation SARS-CoV-2 PCR negative | 0.18 | 0 | 0.52 | 0 |  |  |  |
| 151 | Patient under investigation SARS-CoV-2 PCR negative | 0.18 | 0 | 0.18 | 0 |  |  |  |
| 152 | Dengue IgM/Dengue IgG | 0.53 | 0 | 0.21 | 0 |  |  |  |
| 153 | Dengue IgM | 0.42 | 0 | 0.20 | 0 |  |  |  |
| 154 | Anti-HBS pos | 0.79 | 0 | 0.25 | 0 |  |  |  |
| 155 | Anti-HBS pos | 0.46 | 0 | 0.20 | 0 |  |  |  |
| 156 | Treponemal Ab pos | 0.54 | 0 | 0.18 | 0 |  |  |  |
| 157 | Treponemal Ab pos | 0.62 | 0 | 0.32 | 0 |  |  |  |
| 158 | Anti-HCV pos | 0.80 | 0 | 0.35 | 0 |  |  |  |
| 159 | Anti-HCV pos | 0.36 | 0 | 0.19 | 0 |  |  |  |
| 160 | HBSAg pos | 0.48 | 0 | 0.16 | 0 |  |  |  |
| 161 | HBSAg pos | 0.30 | 0 | 0.18 | 0 |  |  |  |
| 162 | Mumps IgG pos / Measles IgG pos / VZV IgG pos / HSV IgG pos | 0.67 | 0 | 0.27 | 0 |  |  |  |
| 163 | Measles IgG pos / Rubella IgG pos | 0.47 | 0 | 0.18 | 0 |  |  |  |
| 164 | Rubella IgG pos / EBV IgG pos | 0.79 | 0 | 0.27 | 0 |  |  |  |
| 165 | Mumps G pos | 0.77 | 0 | 0.26 | 0 |  |  |  |
| 166 | VZV IgG pos | 0.61 | 0 | 0.22 | 0 |  |  |  |
| 167 | HSV IgM pos | 0.98 | 0 | 0.25 | 0 |  |  |  |
| 168 | CMV IgG pos / CMV IgM pos | >11 | 1 | 2.11 | 1 |  |  |  |
| 169 | CMV IgG pos / CMV IgM pos | 0.49 | 0 | 0.26 | 0 |  |  |  |
| 170 | EBV IgG pos / EBV IgM pos | 10.84 | 1 | 2.35 | 1 |  |  |  |
| 171 | Rubella IgG pos | 0.52 | 0 | 0.18 | 0 |  |  |  |
|  |  | 109.25 |  | 57.39 |  |  |  |  |
|  | Mean | 0.635174419 |  | 0.333662791 |  |  |  |  |
