# Supplementary material for: Antibody responses to SARS-CoV-2 in patients with differing severities of coronavirus disease 2019
Source: PLoS One. 2020 Oct 9;15(10):e0240502. doi: 10.1371/journal.pone.0240502 (PMC7546485; doi:10.1371/journal.pone.0240502)
Supplement: S2 Table — (DOCX) [file pone.0240502.s002.docx]

**Antibody Responses to SARS-CoV-2 in Coronavirus Diseases 2019 Patients with Different Severity**

**S2 Table: Summary serological results from 15 studies based on Euroimmun test**

| References | Sample Tested | Sensitivity | Control | Specificity | Remarks |
| --- | --- | --- | --- | --- | --- |
| Leaflet package of EUROIMMUN  Version: 2020-05-06 | 91 cases (94 samples) | IgA (cut off >1.1)  < 10 days (N=66) = 51.5%  >10-20 days (N=12) = 91.7%  > 21 days (N=16) = 100%  IgG (cut off >1.1)  < 10 days (N=66) = 30.3%  >10-20 days (N=12) = 75%  > 21 days (N=16) = 93.8% | 1241 controls (849 blood donors, 90 children, 200 pregnant women, 40 Influenza, 22 EBV & heterophilic Ab, 40 RF) | IgA (cut off >0.8) = 88.2%  IgA (cut off >1.1) = 92.4%  IgG (cut off >0.8) = 99%  IgG (cut off >1.1) = 99.6% | - Reference Okba et al., 2020 |
| Okba et al., Emerging Infectious Diseases | Netherlands 3 cases (2 mild, 1 severe), 10 serum samples  Berlin 9 cases, 31 serum samples | IgA = 70% (7/10)  IgG = 60% (6/10)  IgA = 88.9% (8/9)  IgG =88.9% (8/9) | 203 controls (45 blood donors, 5 Adenovirus, 2 Bocavirus, 2 Enterovirus, 9 HMPV, 13 Influenza A, 6 Influenza B, 9 Rhinovirus, 9 RSV, 4 PIV-1, 4 PIV-3, 1 Mycoplasma pneumoniae, 5 CMV, 7 EBV, 19 229E, 18 NL63, 38 OC43, 7 MERS-CoV, 2 SARS-CoV)  24 serum samples (4 HCoV-229E, 3 HCoV-HKU1, 7 HCoV-NL634 HCoV-OC43, 3 MERS-CoV, 3 SARS-CoV) | IgA (cut off>0.9) = 94.6% (192/203)  IgG (cut off>0.3) = 96% (195/203)  IgA (cut off>0.9) = 87.5% (21/24)  IgG (cut off>0.3) = 87.5% (21/24) | - Beta-version of EUROIMMUN using in-house cutoff value based on the mean background  - Severe case developed Ab sooner and in higher concentrations.  - Cross-reactivity with HCoV-OC43, SARS-CoV  - Correlation with plaque reduction neutralization assay (PRNT_50_) r =0.5-0.9 |
| Jassskelainen et al., Euro Surveill. | Finland 39 cases (9 mild, 15 moderate, 13 severe, 3 unknown) | Median time after onset of symptoms  IgA = 11 days (5-20 days)  IgG = 12 days (5-20 days) | 37 Cross-reactivity panel: Influenza, Parainfluenza, RSV, Enterovirus, Coronavirus OC43, NL63, 229E | IgA (cut off >1.1) = 73%  IgG (cut off >1.1) = 91.9% | - No correlation with severity was clearly seen.  - IgA cross react with HCoV-OC43 and other respiratory viruses e.g., Influenza, Parainfluenza, RSV, Adenovirus  - IgA was not suggested for initial screening due to low specificity  - IgG cross react with HCoV-OC43  - one case with mild disease severity still has IgG negative 16 days after onset of symptoms |
| Jassskelainen et al., Journal of Clinical Virology | Finland 62 cases (70 samples) | IgA (cut off >1.1)  87.8%  IgG (cut off >1.1)  70.7% | 81 controls (Autoimmune and respiratory virus in 2018 and 2019) | IgA (cut off >1.1)  68.3%  IgG (cut off >1.1)  86.8% | - No correlation between disease severity (N=55) and microneutralization titers  - IgA has 87.8% sensitivity and IgG has 70.7% sensitivity compared with Microneutralization Test  - No cross-react with RF |
| Montesinos et al., Journal of Clinical Virology | Belgium 128 cases | IgA (cut off >0.8)  Overall = 83.6%  <7 days (N=29) = 65.5  8-14 days (N=62) = 87.09%  >15 days (N=33) = 93.93%  IgG (cut off >0.8)  Overall = 61.7%  <7 days (N=29) = 17.2%  8-14 days (N=62) = 66.12%  >15 days (N=33) = 90.9% | 72 controls (5 EBV, 11 CMV, 8 M.pneumoniae, 1 Parvovirus, 1 HBV, 1 Batonella henselae, 1 Brucella spp, 3 autoimmune) | IgA (cut off >0.8) = 86.1%  IgG (cut off >0.8) = 98.6% | - IgA cross-react with EBV, M. pneumoniae, Anti-PL12 and also with healthy control without any known confounding factors. |
| Tang et al., American Association for Clinical Chemistry | USA 48 cases (103 samples)  Hospitalized with multiple comorbidities | IgG (cut off >0.8)  < 3 days (N=12) = 0%  3-7 days (N=20) = 25%  8-13 days (N=23) = 56.5%  >14 days (N=48)= 85.4% | 153 controls (50 serum in 2015, 80 symptomatic but PCR negative for SARS-CoV-2, 5 other coronaviruses, HKU1, NL63, 229E, 4 influenza, 5 CMV, 3 EBV VCA IgG, 3 EBV VCA IgM, 2 EBV IgM and IgG, 1 RF) | IgG (cut off >1.1) = 96.7%  IgG (cut off >0.8) = 94.8% | - 3 of the false positives were from sample collected in 2015.  - 3 patients had no Ab responses at > 14 days post-infection |
| Elslande et al., Clinical Microbiology and Infection | Belgium 94 cases (167 samples) | IgG (cut off >1.1)  14-25 days = 89.5% | 103 controls (49 respiratory infection, 14 PCR negative for SARS-CoV-2, 40 other pathogens e.g., CMV, EBV, HIV) | IgA (cut off >1.1) = 73.8%  IgG (cut off >1.1) = 96.1% | - IgA cross-react with a number of other infections  - Due to low specificity of IgA, not recommend using IgA for screening of asymptomatic persons  - IgG cross react with EBV, CMV, Entero-/Rhinovirus+, HSV1+, Streptococcus pneumoniae+ |
| Kruttgen et al., Journal of Clinical Virology | Germany 31 cases (50 samples) | IgG (cut off >1.1)  7-17 days = 86.4% | 25 controls (PCR negative for SARS-CoV-2) | IgG (cut off >1.1) = 96.2% |  |
| Hardy et al., Clin Chem Lab Med | Belgium 44 cases | IgG (cut off >1.1)  = 96% | 81 controls (1 NL63, 1 OC43, 7 HBV, 3 HAV, 1 Adenovirus, 1 HSV and CMV, 8 CMV, 5 Parvovirus B19, 1 HIV, 4 antistreptolysin O (ASLO), 1 anti Treponema pallidum, 1 Borrelia, 10 Mycoplasma pneumoniae, 16 Toxoplasma gondii, 1 RF, 7 anti-TPO, 4 agglutinins, 1 direct coombs, 1 high level of IgM, 1 high IgA, 6 healthy) | IgG (cut off >1.1) = 98% | - Cross-react with anti-TPO, anti-HAV, ASLO |
| Beavis et al., Journal of Clinical Virology | USA 82 cases | IgA (cut off >1.1) = 82.9%  > 4 days after positive PCR 90.5%  IgG (cut off >1.1) = 67.1%  > 4 days after positive PCR 100% | 86 controls | IgA (cut off >1.1) = 88.4%  IgG (cut off >1.1) = 97.7% | - Borderline cross-react with NL63 and OC43 |
| Nicol et al., Journal of Clinical Virology | France 82 cases (141 samples) | IgA (cut off >1.1)  Overall = 86.7%  <7 days (N=56) = 59.4  8-14 days (N=44) = 79.3%  >15 days (N=98) = 100%  IgG (cut off >0.8)  Overall = 78.3%  <7 days (N=56) = 28.1%  8-14 days (N=44) = 72.4%  >15 days (N=98) = 100% | 155 controls (50 SARS-CoV-2 RT-PCR negative, 50 control before March 2019, 25 cross-reactivity panel, 10 pregnant women, 10 RF) | IgA (cut off >1.1) = 82.7%  IgG (cut off >1.1) = 96.7% | - IgA and IgG cross-react with pregnant serum, RF, patients with symptoms of pneumonia |
| Kohmer et al., Journal of Medical Virology | Germany 33 cases | IgG (cut off >1.1)  5-9 days (N=17) = 58.8%  10-18 days (N=16) = 93.8% | 21 controls (SARS-CoV, OC43, HKU1, NL63, 229E, EBV, CMV) | IgG (cut off >1.1) = 96.2% | - All IgG positive tested samples showed neutralizing properties in the PRNT (tier > 1.20) |
| Kohmer et al., Journal of Clinical Virology | Germany 45 cases | IgG (cut off >1.1)  = 71.1% | 22 controls (SARS-CoV, OC43, HKU1, NL63, 229E, EBV, CMV) | IgG (cut off >1.1) = 100% | - Equivocal results with OC43 and negative control cohort)  - Correlate well with PRNT |
| Theel et al., Journal of Clinical Microbiology | USA 56 cases (224 samples from 33 in-patient and 23 out-patient) | IgG (cut off >1.1)  <7 day (N=38) = 0 (in-patient), (N=11) = 18.2% (out-patient)  8-14 days (N=91) = 27.5% (in-patient)  >15 days (N=61) =100% (in-patient)  > 20 days (N=23) = 91.3% (out-patient) | 149 controls from 2018  105 cross-reactivity panel (CMV, Influenza, Mycoplasma pneumoniae, Chlamydophila pneumoniae, Streptococcus pneumoniae urinary antigen, coronavirus, metapneumovirus, RSV, adenovirus, rhinovirus/enterovirus, HBV, HCV, HIV) | IgG (cut off >1.1)  99.3% (healthy)  96.2% (Cross-reactivity panel)  98% (overall) |  |
| Tuaillon et al., Journal of Infection | France 38 cases | IgA (cut off >1.1)  93.3%  IgG (cut off >1.1)  93.3% | 20 controls | IgA (cut off >1.1)  80%  IgG (cut off >1.1)  85% |  |

References

1. Euroimmun [package insert]. Anti-SARS-CoV-2 ELISA (IgA and IgG) In: Companies AP, editor. 2020.Euroimmun. Germany: A PerkinElmer Companies; Version: 2020-05-06.

2. Okba NMA, Muller MA, Li W, Wang C, GeurtsvanKessel CH, Corman VM, et al. Severe Acute Respiratory Syndrome Coronavirus 2-Specific Antibody Responses in Coronavirus Disease Patients. Emerg Infect Dis. 2020;26(7):1478-88.

3. Jääskeläinen AJ, Kekäläinen E, Kallio-Kokko H, Mannonen L, Kortela E, Vapalahti O, et al. Evaluation of commercial and automated SARS-CoV-2 IgG and IgA ELISAs using coronavirus disease (COVID-19) patient samples. Euro Surveill. 2020;25(18).

4. Jaaskelainen AJ, Kuivanen S, Kekalainen E, Ahava MJ, Loginov R, Kallio-Kokko H, et al. Performance of six SARS-CoV-2 immunoassays in comparison with microneutralisation. J Clin Virol. 2020;129:104512.

5. Montesinos I, Gruson D, Kabamba B, Dahma H, Van den Wijngaert S, Reza S, et al. Evaluation of two automated and three rapid lateral flow immunoassays for the detection of anti-SARS-CoV-2 antibodies. J Clin Virol. 2020;128:104413.

6. Tang MS, Hock KG, Logsdon NM, Hayes JE, Gronowski AM, Anderson NW, et al. Clinical Performance of Two SARS-CoV-2 Serologic Assays. Clin Chem. 2020.

7. Van Elslande J, Houben E, Depypere M, Brackenier A, Desmet S, Andre E, et al. Diagnostic performance of seven rapid IgG/IgM antibody tests and the Euroimmun IgA/IgG ELISA in COVID-19 patients. Clin Microbiol Infect. 2020.

8. Krüttgen A, Cornelissen CG, Dreher M, Hornef M, Imöhl M, Kleines M. Comparison of four new commercial serologic assays for determination of SARS-CoV-2 IgG. J Clin Virol. 2020;128:104394.

9. Tre-Hardy M, Wilmet A, Beukinga I, Dogne JM, Douxfils J, Blairon L. Validation of a chemiluminescent assay for specific SARS-CoV-2 antibody. Clin Chem Lab Med. 2020;58(8):1357-64.

23.

10. Beavis KG, Matushek SM, Abeleda APF, Bethel C, Hunt C, Gillen S, et al. Evaluation of the EUROIMMUN Anti-SARS-CoV-2 ELISA Assay for detection of IgA and IgG antibodies. J Clin Virol. 2020;129:104468.

11. Nicol T, Lefeuvre C, Serri O, Pivert A, Joubaud F, Dubée V, et al. Assessment of SARS-CoV-2 serological tests for the diagnosis of COVID-19 through the evaluation of three immunoassays: Two automated immunoassays (Euroimmun and Abbott) and one rapid lateral flow immunoassay (NG Biotech). J Clin Virol. 2020;129:104511.

12. Kohmer N, Westhaus S, Rühl C, Ciesek S, Rabenau HF. Clinical performance of different SARS-CoV-2 IgG antibody tests. J Med Virol. 2020.

13. Kohmer N, Westhaus S, Ruhl C, Ciesek S, Rabenau HF. Brief clinical evaluation of six high-throughput SARS-CoV-2 IgG antibody assays. J Clin Virol. 2020;129:104480.

14. Theel ES, Harring J, Hilgart H, Granger D. Performance Characteristics of Four High-Throughput Immunoassays for Detection of IgG Antibodies against SARS-CoV-2. J Clin Microbiol. 2020.

15. Tuaillon E, Bolloré K, Pisoni A, Debiesse S, Renault C, Marie S, et al. Detection of SARS-CoV-2 antibodies using commercial assays and seroconversion patterns in hospitalized patients. The Journal of infection. 2020.
