# Supplementary material for: Antibody responses to SARS-CoV-2 in patients with differing severities of coronavirus disease 2019
Source: PLoS One. 2020 Oct 9;15(10):e0240502. doi: 10.1371/journal.pone.0240502 (PMC7546485; doi:10.1371/journal.pone.0240502)
Supplement: Attachment — Submitted filename: review.docx [file pone.0240502.s003.docx]

This is a well written paper that I will be of use to clinicians and laboratory scientist dealing with the COVID-19 pandemic. The study is largely descriptive but is comprehensive and the information regarding days of illness, age groups and diagnostic accuracy of the Euroimmun SARS-CoV-2 ELISA is useful.

There are some problems with the English and the manuscript would benefit from an edit by a native English language speaker.

I have some questions relating to this manuscript which I would appreciate if the authors can respond.

1. Can the authors please explain why they did not test for IgM?

2. Why did the authors choose to use their own severity score when there are already multiple severity scores available. Can the authors please comment on the limitations relating to the use of their own severity score.

A comparison of COVID-19 severity scores is contained in the following citation. Guohui Fan, Chao Tu, Fei Zhou, Zhibo Liu, Yeming Wang, Bin Song, Xiaoying Gu, Yimin Wang, Yuan Wei, Hui Li, Xudong Wu, Jiuyang Xu, Shengjin Tu, Yi Zhang, Wenjuan Wu, Bin Cao European Respiratory Journal 2020; **DOI:** 10.1183/13993003.02113-2020

3. The authors note that they used both plasma and serum for this study. Is there any difference between plasma and serum in the ELISA.

4. The false positives are interesting. In line 177 the authors state “From 20 serum specimens  collected from patients with other infections revealed two samples with both IgA and IgG cross-reactivity with CMV and EBV positive samples”. Can the authors prove that this is cross-reactivity or is it true positivity due to across reaction with another coronavirus?

5. In line 175 the authors state “It should be noted that the positive results in these patients might be the result of either false positive or true positive cases with negative RT-PCR. However, we did not have any evidence to support the COVID-19 infection in these patients”. This is conjecture and should be moved to the discussion section.

6. Given the issues relating to cross-reactivity or false positivity with SARS-CoV-2 serology – would it be possible to provide more information regarding the false positive results. The information provided is inadequate.
